# Two Views of What the Dental Society Meeting Should Be

**Published:** 1913-12-15

**Authors:** 


### TWO VIEWS OF WHAT THE DENTAL SOCIETY MEETING SHOULD BE.

BY TWO EDITORS.

"Largely Attended Dental Meetings not Necessarily Successful Ones." Attendance upon dental society meetings during the past decade has revealed to the minds of many members thereof the regrettable fact that, with all our finely illustrated scientific papers, increased clinics, magnificent exhibits, etc., there has not been a corresponding increase in attendance upon the distinctly educational features of such meetings. Those who have closely observed conditions have no hesitancy in saying that there exists a very visible apathy toward that portion of the meetings which should be most generously patronized, *i. e.*, the sections devoted to the reading of essays and presentation of scientific reports. A comparison of programs of present-day dental society meetings with those held ten or fifteen years ago shows that, while the dental gathering of to-day—be it National, State or local in character—presents excellent papers and clinics, it also contains a greater percentage of factors tending to disconcert the members in attendance from the purpose for which every dental meeting is or should be held—that of instructing and broadening in the knowledge of dental subjects.

There is without question a tendency on the part of many of those having dental meetings in charge to consider a large attendance the chief requisite of a successful dental gathering, and in order to produce this result various inducements in the way of entertainment are held out to the members. In fact, this is often made so apparent that the prevailing idea in many men's minds seems to be the having of a good time, with a half day devoted to watching clinicians, and frequent visits to the exhibitors quarters. The essays and reports of standing committees are regarded as something to be carefully avoided by a large proportion of this present-day increased attendance, with the excuse, if one be asked, that the meeting room is hot and stuffy, and "besides, we can read the papers when published in the journals or proceedings."

If anyone doubt whether this condition of affairs exists, let him compare the attendance upon the reading of papers in meetings of say only ten years ago with those of the present day, and then consider that the membership in most societies, National and State, has in most instances doubled or trebled. It would seem, then, that either the scientific paper of to-day has decreased in worth or that a greater percentage of the attendants belongs to that unfortunate portion of the profession which feels that it knows all that is necessary or demamded of it.

We do not feel that we are taking a too pesimistic view in expressing these thoughts regarding dental society conditions. In fact, we are quite sure that never before has the profession been given more scientific and practical papers than it has in the meetings of to-day. But we do feel that the annual dental gathering of the present day has unfortunately and unconsciously degenerated; that the meeting of to-day is in many respects inferior to that of some years ago, when bodies of dignified gentlemen met with the one desire in their minds—to learn, and incidentally to teach.

There may be—doubtless are—a number of reasons for the present scientific apathy mentioned, and possibly this condition appears exaggerated, due to the large numbers who attend meetings and conscientiously circulate in every other portion of the meeting except the convention hall. But to the writer it would seem a wise step to eliminate from our programs all attractions not dental in character. What if our attendance does decrease? Only those who are of little benefit to the society, or the society to them, would remain away, and that might prove beneficial to all parties concerned. After a short time many of these would doubtless wake up to find themselves creeping behind, and would fall in line again to keep up with their professional brothers.

Another feature which should be observed is the closing of manufacturer's exhibits during the hours scheduled on the programs for essays, reports and clinics. In many places this is done, and almost invariably proves advantageous to both dentist and exhibitors.

If, instead of our committees making efforts by offering extraneous attractions to have attendances of 300 or 400 at the meetings, regardless of the quality of such attendance, they should endeavor to bring together the workers and "good listeners"—those actually interested in the furtherance of dental science—they would not only produce more dignified, scientific and beneficial meetings, but rid themselves of a deal of unnecessary hustling and brain-conjuring in trying to provide something that would "draw the crowds."—Editorial in September *Brief*.

---

The September number of *The Dental Brief* contains an editorial, "Largely Attended Dental Meetings Not Necessarily Successful Ones."—Quoted above.—The editor thinks the modern dental meeting is degenerating to clinics, entertainments, exhibits, etc.

It is our opinion that the average dental meeting of to-day is far away and ahead of that of even ten years ago. The newer generation of dentists are better prepared, better educated. The papers are more intelligently discussed and of a higher character. This, of course, because of the higher educational standards of our dental colleges. Dentistry has been blessed with men of good education and high standards but we are speaking of the average dentist. The small meeting means that you have to force men from the clinics and exhibits to get a decent audience to listen to the papers and in the case of a distinguished invited guest, this is at times embarrassing.

With some dentists, the dental meeting is their only vacation and they are not all interested in the scientific paper that will later appear in the dental journal. The average paper is of too great length and too little time is given to the discussion and when discussed it is too frequently by little Willie Spot Light who wants to get in the printed discussion. (When the editor sends him his proof he re-writes the whole business and tries to say all the things he didn't say and should have said.)

Little Willie suffers from a diarrhoea of words and a constipation of ideas and his talk reminds you of a cup of cold water swashing around in a gas tank. About the time he gets through, the discussion is called for lack of time and the necessity of presenting the next paper.

With the big meeting you let the man who is out for a good time arrange his own schedule, also the man who wants to get a college education with his hands and fingers.

If he is from the country district this may be his only opportunity for intelligent comparison and advice in the purchase of a much-needed cabinet or engine. There are enough left to at least partially fill the meeting room and listen to the papers.

Then there is the good fellowship, the meeting of old college mates, who discuss the paper and damn the essayist with faint praise, but none of this gets in the printed proceedings. They swap lies and tell of a new wrinkle that has helped them in practice.

At a recent meeting of the Seventh and Eighth District Dental Societies, there were present over 200 graduates from a certain college and they had a banquet. It was said to be the largest number of graduates from this institution ever gotten together. Did they have a good time? Was it a good meeting? The best ever. Also every man carried home some new ideas.

No, Buddy, you're in wrong. Let's have the big meetings. The telephone, stationery, typewriter, ice water, the rugs, palms, and flowers. The "Hail, fellow, well met," the banquet, theater and taxicab drive and all the information we can get without injury. He works best who plays best and meanwhile you and I, who want the intellectual feast, and elbow with the great and near great, can join in the fun between times.—Editorial in September *Dispensary Record.*

